# The PLANES model for unresectable hepatocellular carcinoma treated with transcatheter arterial chemoembolization plus lenvatinib and PD-1 inhibitors: a multicenter, retrospective study

**DOI:** 10.3389/fimmu.2025.1680956

**Published:** 2026-01-15

**Authors:** Houxiang Ya, Kai Wang, Jiayi Wu, Junyi Wu, Zhenxin Zeng, Yinan Li, Xiangye Ou, Huachun Song, Maolin Yan, Shuqun Li

**Affiliations:** 1 Department of Hepatobiliary Pancreatic Surgery, The First Affiliated Hospital of Guilin Medical University, Guilin, Guangxi, China; 2 Department of Hepatopancreatobiliary Surgery, Affiliated Hospital Shenzhen Baoan Central Hospital of Guangdong Medical University, Shenzhen, Guangdong, China; 3 Department of Hepatobiliary Pancreatic Surgery, Fujian Provincial Hospital, Fuzhou, Fujian, China

**Keywords:** lenvatinib, nomogram, PD-1 inhibitors, TACE, unresectable hepatocellular

## Abstract

**Background:**

The triple therapy regimen of transcatheter arterial chemoembolization (TACE) plus lenvatinib and PD-1 inhibitors plays a significant role in the treatment of unresectable hepatocellular carcinoma (uHCC). However, only a subset of patients can benefit from it, making it crucial to screen for those who may benefit from the triple therapy regimen. This study constructed and assessed a survival prediction nomogram for uHCC patients undergoing first-line triple therapy.

**Methods:**

Using retrospective data from 277 consecutive triple-therapy patients (treated at six Chinese centers between 2018–2023), we constructed the nomogram based on multivariate-derived predictors. Model performance was quantified through discrimination metrics and bootstrapped internal validation. External validation employed an independent cohort (n=208) from three additional institutions.

**Results:**

Independent predictors of overall survival (OS) included alpha-fetoprotein (AFP), maximum tumor diameter, tumor number, extrahepatic metastasis, and platelet-to-lymphocyte ratio (PLR). A PLANES model was constructed to predict 12-, 24-, and 36-month OS. The model demonstrated robust discriminative performance, with area under the curve (AUC) values of 0.887, 0.793 and 0.749 for 12-, 24-, and 36-month OS in the training cohort, respectively. External validation yielded AUCs of 0.922, 0.760, and 0.722 for corresponding time points.

**Conclusions:**

The PLANES model was successfully established and validated for predict prognosis in unresectable HCC patients receiving first-line triple therapy.

## 1 Introduction

Hepatocellular carcinoma (HCC) is a notably fatal and prevalent malignancy. Radical resection, ablation and liver transplantation are considered curative therapy. Unfortunately, most cases are un-resectable HCC (uHCC) due to its insidious onset and thus have a poor prognosis (1). With the progression of systemic therapies including molecularly targeted agents, especially the emergence of immune checkpoint inhibitors (ICIs), the treatment for uHCC have greatly improved (2–5). Furthermore, recent studies have underscored the limitations of traditional staging systems and begun to explore novel prognostic factors for HCC (6–8). Nevertheless, monotherapy regimens yield suboptimal outcomes, with clinical studies reporting median progression-free survival (mPFS) of 2.1–7.3 months and median overall survival (mOS) of 6.4–22.1 months (9–13). A number of combination systemic therapeutic strategies have been investigated for patients with uHCC and improving the efficacy of these patients. To enhance efficacy, combination strategies have been explored. The LEAP-002 demonstrated a mOS of 21.1 months with lenvatinib plus pembrolizumab (14), while the phase III IMbrave150 trial established significant OS/PFS benefits of atezolizumab-bevacizumab versus sorafenib (15). Similarly, the phase III HIMALAYA trial reported 18- and 24-month survival rates of 48.7% and 40.5% with durvalumab-tremelimumab (16). Among the systemic therapies, combining tyrosine kinase inhibitors (TKIs) with ICIs exhibited an impressive findings.

For intermediate and advanced HCC, transcatheter arterial chemoembolization (TACE) constitutes guideline-based management widely adopted in clinical practice (17–21). However, TACE monotherapy has limited efficacy. Clinical studies now explore TACE combined with systemic agents: A nationwide real-world analysis confirmed that TACE plus programmed death-(ligand)1 (PD-[L]1) inhibitors and molecular targeted therapies (MTT) improves objective response rate (ORR), OS, and PFS in uHCC (22). A systematic review further established the superiority of TACE combined with TKIs and ICIs over TACE+TKIs, TKIs+ICIs, or TKI monotherapy in conversion rates, ORR, disease control rate (DCR), PFS, and OS (23). Another review—including our prior retrospective study—reported triple therapy (TACE + lenvatinib + anti-PD-1 antibodies) achieving ORR of 26.1–87.2%, DCR of 70–100%, median PFS of 6.3–22.5 months, and median OS of 15.7–29 months (24).

Despite these promising outcomes, disease progression remains common, and large-scale studies identifying optimal beneficiaries of triple therapy are lacking. This multicenter retrospective study therefore aims to develop and validate a prognostic model to identify uHCC patients most likely to benefit from triple therapy. This manuscript is written following the TRIPOD checklist.

## 2 Methods

### 2.1 Patients

This multicenter retrospective analysis enrolled 576 consecutive patients with uHCC initiating first-line triple therapy (TACE + lenvatinib + PD-1 inhibitors) between June 2018 and December 2023 across six tertiary medical centers in China. The training cohort included 277 patients from the Fujian Provincial Hospital, the Second Affiliated Hospital of Nanchang University and the First Affiliated Hospital of Guilin Medical University. The external validation cohort included 208 patients from the First Affiliated Hospital of Xiamen University, Eastern Hepatobiliary Surgery Hospital and Zhangzhou Affiliated Hospital of Fujian Medical University. This study was conducted following the principle of the Declaration of Helsinki.

All HCC diagnoses adhered to the China Liver Cancer Staging criteria (25), with tumor unresectability confirmed through multidisciplinary team (MDT) consensus. Inclusion criteria required (1): age 18–75 years (2), diagnosed as oncologically unresectable with first-line triple therapy initiation (3), Eastern Cooperative Oncology Group (ECOG) performance status 0-1 (4), Child–Pugh class A or B and BCLC stage B or C, and (5) ≥1 measurable lesion per mRECIST. Exclusion criteria comprised (1): Child–Pugh class C (2), prior receipt of systemic therapy or interventional therapy for HCC (3), concurrent malignancies (4), active autoimmune disorders or immunodeficiency (5), Death within 3 months, and (6) missing important data.

### 2.2 Treatment strategies

All TACE procedures adhered to standardized protocols established across participating hospitals (26, 27). These procedures were conducted exclusively by physicians with a minimum of ten years’ experience in interventional radiology, drawn from the collaborating centers. Each patient included in this study underwent conventional TACE. Superselective TACE was performed by specialists who had at least five years of experience. The number, position, size, and supplying arteries of the tumors were determined by angiography. A mixed emulsion of iodide oil (5–20 mL) and pirarubicin infused into the tumor supplying arteries. At last, the supplying arteries blood flow was interrupted by gelatin sponge granules embolization. TACE was repeated at 4–6 week intervals for patients with residual viable tumors or new intra-hepatic lesions identified on follow-up imaging, provided their liver function remained adequate.

Lenvatinib was initiated within 3–14 days post-initial TACE using weight-based dosing (8 mg/day for <60 kg; 12 mg/day for ≥60 kg). In the event of Grade 1–2 adverse events (AEs), maintain the current dose and promptly administer supportive care to manage these events. For Grade 3–4 adverse events, reduce the dose to 8 mg and 4 mg, respectively, or adjust the dosing frequency to every other day until the adverse events are alleviated or improved. For persistent adverse events, suspend medication until the events are resolved or have disappeared. PD-1 inhibitors (camrelizumab 200 mg, tislelizumab 200 mg, sintilimab 200 mg, toripalimab 240 mg, pembrolizumab 200 mg, or penpulimab 200 mg) were administered intravenously every 3 weeks. Both agents were suspended 3 days pre-TACE and resumed 3 days post-procedure absent severe complications. Chronic hepatitis B virus (HBV) carriers received entecavir or tenofovir antiviral prophylaxis.

### 2.3 Study design

Data on consecutive patients with uHCC who underwent triple therapy as an initial treatment were included. All variables were collected at baseline prior to the commencement of any treatment. In this study, OS is defined as the time period from enrollment to death from any cause or the last follow-up. Tumor response was evaluated per mRECIST criteria (28) via contrast-enhanced CT/MRI, classifying outcomes as complete (CR), partial (PR), stable (SD), or progressive disease (PD). ORR and DCR were calculated as (CR+PR)/total and (CR+PR+SD)/total, respectively.

The following variables are included: sex, age, Barcelona Clinic Liver Cancer (BCLC) stage, Child-Pugh stage, Eastern Cooperative Oncology Group performance status (ECOG PS) score, alpha- fetoprotein (AFP) levels, HBV infection, maximum tumor size, tumor number, macrovascular invasion (MVI), extra-hepatic metastasis (EHM), neutrophil-to-lymphocyte ratio (NLR), platelet-to-lymphocyte ratio (PLR), albumin-bilirubin ratio (ALBI) grade, alanine transaminase (ALT) levels, aspartate transaminase (AST) levels and other relevant factors. Treatment-related adverse events (TRAEs) were graded using CTCAE v5.0. Patients underwent clinical/imaging assessments every 4–8 weeks. For patients achieving resectability, lenvatinib was withheld 1 week and PD-1 inhibitors 4 weeks before surgery. Therapy discontinuation occurred upon disease progression, unacceptable toxicity, or patient withdrawal.

### 2.4 Follow-up

Patients underwent clinical assessments at 4-8-week intervals. Surgical intervention was pursued when tumors fulfilled R0 resection criteria with sufficient hepatic remnant volume. Perioperatively, we suspended lenvatinib for 1 week and PD-1 inhibitors for 4 weeks. Postoperative systemic therapy continued for 3–12 months based on histopathological and imaging evaluations. For non-surgical patients, triple therapy cessation occurred upon disease progression, intolerable adverse events, or patient withdrawal. Subsequent management followed multidisciplinary team consensus and patient consultation.

### 2.5 Statistical analysis

IBM SPSS Statistics (v26.0) and R (v4.2.2) facilitated all analyses. Continuous variables were compared via Mann-Whitney U or Student’s t-tests (expressed as mean ± SD or median[IQR]), while categorical variables used χ² or Wilcoxon rank-sum tests (reported as frequencies/percentages). Continuous variables were dichotomized based on established clinical thresholds or statistical methods. Specifically, the cut-off values for AFP, tumor size, ALT, and AST were selected based on their widespread use in prior literature on HCC (29, 30). For NLR and PLR, the optimal cut-off values for predicting overall survival were determined using receiver operating characteristic (ROC) curve analysis, maximizing Youden’s index. The ALBI score was computed as previously described (31) and categorized into ‘ALBI Low’ (score < -2.60) and ‘ALBI High’ (score ≥ -2.60) groups. OS curves employed Kaplan-Meier methodology with log-rank testing. Univariate Cox regression identified potential OS predictors (p<0.05 threshold), with significant variables entering multivariate Cox proportional hazards modeling (backward stepwise selection). The resultant PLANES nomogram underwent discrimination validation via Harrell’s C-index and time-dependent AUCs. Calibration plots compared predicted versus observed survival probabilities. External validation cohorts replicated these analyses, clinical applicability was evaluated via decision curve analysis (DCA). Missing data were managed through single and multiple imputation techniques. All statistical tests were two-sided with P = 0.05 significance level.

## 3 Results

### 3.1 Baseline characteristics

**Figure 1:**
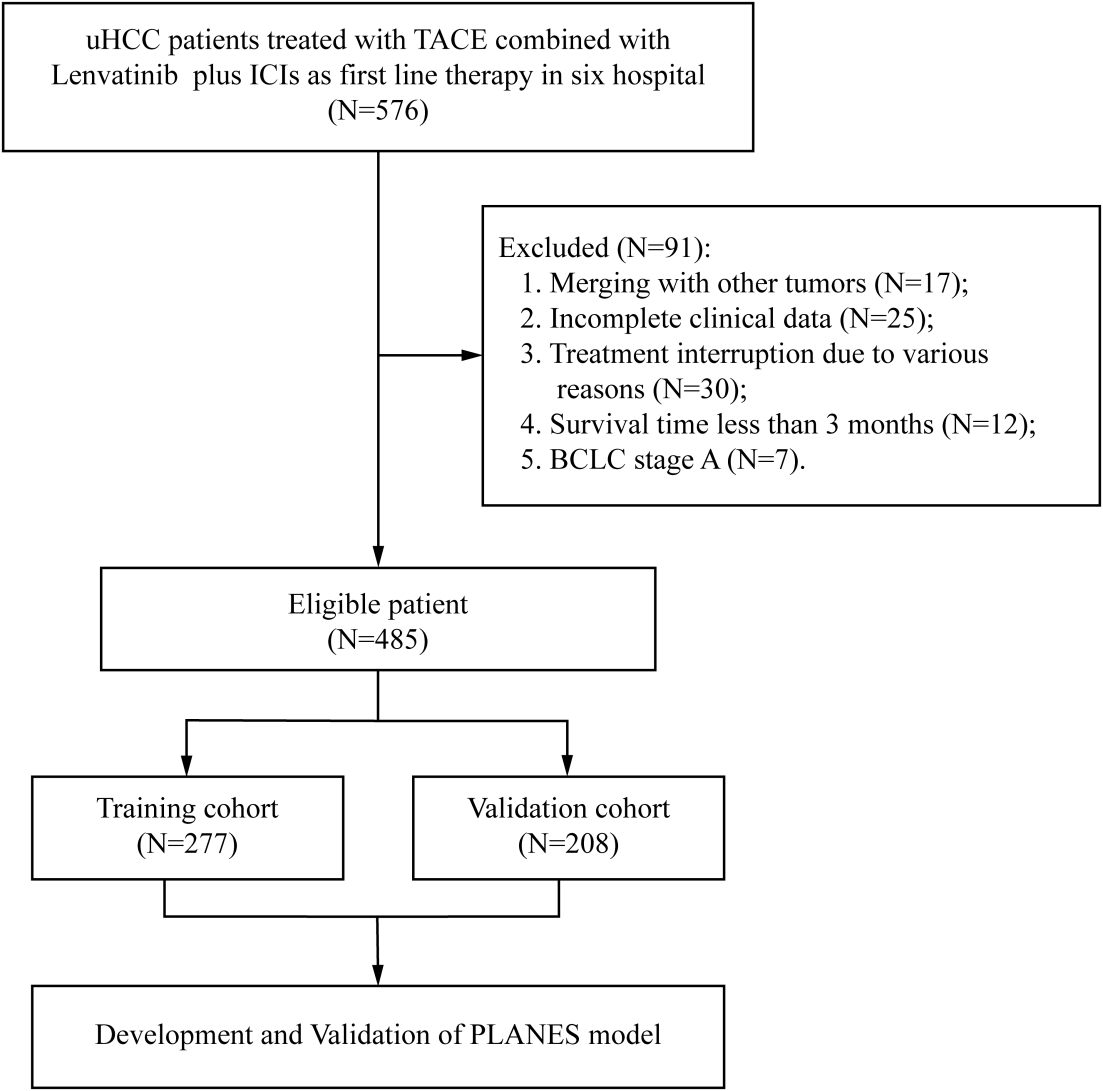
Flowchart of the patient enrollment process for the training cohort and validation cohort.

485 consecutively enrolled uHCC patients receiving first-line triple therapy were screened from six hospitals (**Figure 1**). The cohort comprised 414 males and 71 females. The majority of patients were <65 years old. A total of 368 cases (75.9%) had an ECOG-PS score of 0. Most cases were infected with hepatitis B. Patients with BCLC stage B and stage C accounted for 59.8% and 40.2%, respectively. A total of 140 patients (28.9%) were complicated with MVI, and 253 cases (52.2%) had AFP greater than 400 ng/ml. There were 416 patients (85.8%) with multiple tumors, and 350 patients (72.2%) had the Maximum tumor size≥5 cm. Baseline characteristics of training (n=277) and validation (n=208) cohorts are detailed in **Table 1**.

**Table 1:** Baseline demographic and clinical characteristics of two cohorts.

Variables	Training cohort (n=277)	Validation cohort (n=208)	P value
Sex, n(%)			0.785
Female	39 (14.1%)	32 (15.4%)	
Male	238 (85.9%)	176 (84.6%)	
Age, n(%)			0.753
<65	218 (78.7%)	167 (80.3%)	
≥65	59 (21.3%)	41 (19.7%)	
BCLC stage, n(%)			0.87
B	165 (59.6%)	125 (60.1%)	
C	112 (40.4%)	83(39.9%)	
Child-Pugh stage, n(%)			1
A	248 (89.5%)	187 (89.9%)	
B	29 (10.5%)	21 (10.1%)	
ECOG PS, n(%)			0.61
0	213 (78.0%)	155 (75.6%)	
1	60 (22.0%)	50 (24.4%)	
AFP, n(%)			0.582
<400ng/ml	136 (49.1%)	96 (46.2%)	
≥400ng/ml	141 (50.9%)	112 (53.8%)	
HBV infection, n(%)			0.522
Absence	30 (10.8%)	18 (8.65%)	
Presence	247 (89.2%)	190 (91.3%)	
Maximum tumor size, n(%)			0.536
<5cm	76 (27.4%)	59(28.3%)	
≥5cm	201 (72.64%)	149 (71.7%)	
Tumor number, n(%)			0.144
Single	41 (14.8%)	28 (13.4%)	
Multiple	236 (85.2%)	180 (86.6%)	
MVI, n(%)			0.158
No	201(73.6%)	144(69.6%)	
Yes	76(26.4%)	63(30.4%)	
EHM, n(%)			0.365
No	225(81.2%)	188(77.4%)	
Yes	52(18.8%)	47(22.6%)	
Type of ICIs, n(%)			0.617
Carilizumab	43(15.5%)	28(13.5%)	
Sintilimab	32(11.6%)	31(14.9%)	
Tislelizumab	76(27.4%)	48(23.1%)	
Tremelizumab	29(10.4%)	26(12.5%)	
Penpulimab	27(9.7%)	18(8.6%)	
Pembrolizumab	70(25.4%)	57(27.4%)	
NLR	2.72 (1.83-4.83)	2.64 (1.86-4.71)	0.856
<2.8	182(65.7%)	128(61.5%)	
≥2.8	95(34.3%)	80(39.5%)	
PLR	127 (87.8-185)	125 (85.5-189)	0.973
<175	160(57.8%)	125(60.1%)	
≥175	117(42.2%)	83(39.9%)	
ALBI grade, n(%)			1
High	133 (48.0%)	100(48.1%)	
Low	141 (50.9%)	108 (51.9%)	
ALT, n(%)			0.564
<40(U/L)	139 (50.2%)	98 (47.1%)	
≥40(U/L)	138 (49.8%)	110 (52.9%)	
AST, n(%)			0.393
<40(U/L)	87 (31.4%)	57 (27.4%)	
≥40(U/L)	190 (68.6%)	151 (72.6%)	

BCLC, Barcelona Clinic Liver Cancer; ECOG-PS score, Eastern Cooperative Oncology Group performance status score; AFP, alpha-fetoprotein; MVI, macrovascular invasion; EHM, extra-hepatic metastasis. ICIs, immune checkpoint inhibitors; NLR, neutrophil-to-lymphocyte ratio; PLR, platelet-to-lymphocyte ratio; ALBI: albumin-bilirubin ratio; ALT, alanine transaminase; AST, aspartate transaminase.

### 3.2 Treatment efficacy and safety

Best tumor responses per mRECIST criteria are summarized in **Table 2**: 74 patients (15.2%) achieved CR, 220 (45.4%) achieved PR, 94 (19.4%) achieved SD, and 97 (20.0%) achieved PD. This yielded an ORR of 60.6% and DCR of 80.0%. 87 patients (17.9%) in our cohort underwent conversion surgery with curative intent following triple therapy.

**Table 2:** Tumor responses per investigator assessment (mRECIST).

Best responses	Total (n=485)
CR	74 (15.2%)
PR	220 (45.4%)
SD	94 (19.4%)
PD	97 (20%)
ORR	60.6%
DCR	80.0%

CR, complete response; PR, partial response; SD, stable disease; PD, progressive disease; ORR, objective response rate; DCR, Disease control rate.

TRAEs occurred in 232/277 patients (83.8%) in the training cohort. Most common were: abnormal liver function (62.5%), fever (30.7%), hypertension (27.4%), fatigue (23.5%), decreased appetite (23.1%), and nausea (17.7%). Most events were grade 1-2; no treatment-related deaths occurred. Grade 4 TRAEs developed in 12 patients (4.3%), all resolved after lenvatinib dose reduction (**Table 3**).

**Table 3:** Treatment-related adverse events in training cohort.

Adverse event	n(%)			
	Any grades	Grades 1 and 2	Grades 3	Grades 4
Total	232(83.8)	169(61.1)	51(18.4)	12(4.3)
Fatigue	65(23.5)	55(19.9)	10(3.6)	–
Decreased appetite	64(23.1)	49(17.7)	14(5.1)	–
Fever	85(30.7)	82(29.6)	3(1.1)	–
Nausea	49(17.7)	39(14.1)	10(3.6)	–
Vomiting	30(10.8)	25 (9)	5(1.8)	–
Abdominal pain	33(11.9)	30(10.8)	3(1.1)	–
Hand-foot Syndrome	39(14.1)	31(11.2)	6(2.2)	2(0.7)
Diarrhea	34(12.3)	34(12.3)	–	–
Hypertension	76(27.4)	59(21.3)	17(6.1)	–
Proteinuria	30(10.8)	19(6.9)	9(3.2)	2(0.7)
Rash	31(11.2)	21(7.6)	7(2.5)	3(1.1)
Thrombocytopenia	39(14.1)	39(14.1)	–	–
Hypothyroidism	46(16.6)	36 (13)	10(3.6)	–
Abnormal liver function	173(62.5)	141(50.9)	27(9.7)	5(1.8)

### 3.3 Survival analysis

**Figure 2:**
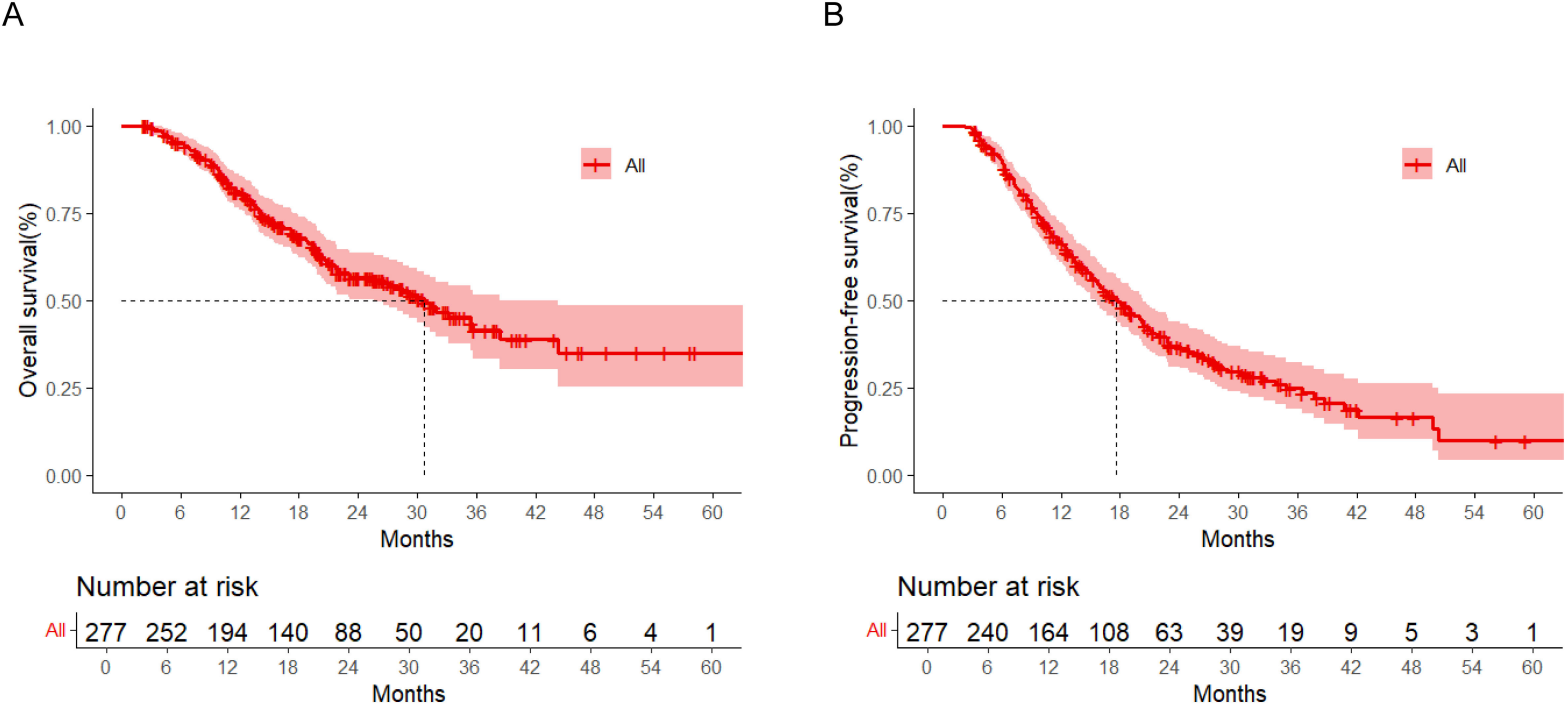
Kaplan-Meier analysis of the overall survival **(A)** and progression-free survival **(B)** in the training cohort.

The training cohort demonstrated a mOS of 30.8 months (95% confidence interval (CI): 4.6-51.3), with corresponding 1-/2-/3-year survival rates of 70.0%, 52.2%, and 40.3%. The mPFS reached 17.8 months (95% CI: 2.5-51.3). In the validation set, the mOS and mPFS were 29.3 months and 15.4 months, respectively. At data cutoff, median follow-up duration was 25.6 months (95% CI: 11.3-43.5) (**Figure 2**).

### 3.4 Prognostic factor analysis

Univariate Cox analysis identified seven OS predictors: ECOG-PS, AFP, maximum tumor diameter, tumor number, MVI, EHM, and PLR (**Table 4**). Multivariate analysis confirmed five independent prognostic factors: AFP (HR: 1.710, 95% CI: 1.071-2.728), maximum tumor diameter (HR: 1.523, 95% CI: 0.912-2.731), tumor number (HR: 1.325, 95% CI: 0.587-2.658), PLR (HR: 1.426, 95% CI: 0.842-2.624), and EHM (HR: 1.720, 95% CI: 1.060-2.794).

**Table 4:** Univariate and Multivariate Cox regression analyses for OS in the training cohort.

Variables	Univariate analysis			Multivariate analysis		
	HR	95% CI	P valuer	HR	95% CI	P value
Gender (male/female)	1.015	0.513–2.029	0.966			
Age (≥/<65 year)	1.198	0.700–2.052	0.509			
BCLC stage (B/C)	0.968	0.679-3.678	0.654			
Child-Pugh (B/A)	0.635	0.432-1.752	0.531			
ECOG PS (0/1)	0.968	0.833–2.252	0.020	0.645	0.359-3.674	0.128
AFP (≥/<400 ng/ml)	1.955	1.234–3.100	0.004	1.710	1.071–2.728	0.024
HBV infection(Presence/Absence)	0.798	0.427–1.480	0.472			
Maximum tumor diameter (≥/<5cm)	1.246	0.800–1.939	0.032	1.523	0.912-2.731	0.031
Tumor number (Multiple/Single)	1.121	0.662–1.887	0.041	1.325	0.587-2.658	0.035
MVI (Yes/No)	1.488	0.943–2.352	0.029	1.267	0.792–2.025	0.324
EHM (Yes/No)	2.784	1.100–3.878	0.018	1.720	1.060–2.794	0.028
ICIs	0.839	0.673-1.295	0.494			
NLR (≥/<2.8)	1.934	1.230–3.034	0.152			
PLR (≥/<175)	1.361	0.881–2.109	0.004	1.426	0.842-2.624	0.043
ALBI grade (High/Low)	1.438	0.910–2.274	0.118			
ALT(≥/<40U/l)	0.810	0.516–1.264	0.352			
AST (≥/<40U/l)	1.363	0.833–2.230	0.219			

BCLC, Barcelona Clinic Liver Cancer; ECOG-PS score, Eastern Cooperative Oncology Group performance status score; AFP, alpha- fetoprotein; MVI, macrovascular invasion; EHM, extra-hepatic metastasis. ICIs, immune checkpoint inhibitors; NLR, neutrophil-to-lymphocyte ratio; PLR, platelet-to-lymphocyte ratio; ALBI: albumin-bilirubin ratio; ALT, alanine transaminase; AST, aspartate transaminase.

### 3.5 Development and validation of PLANES model

**Figure 3:**
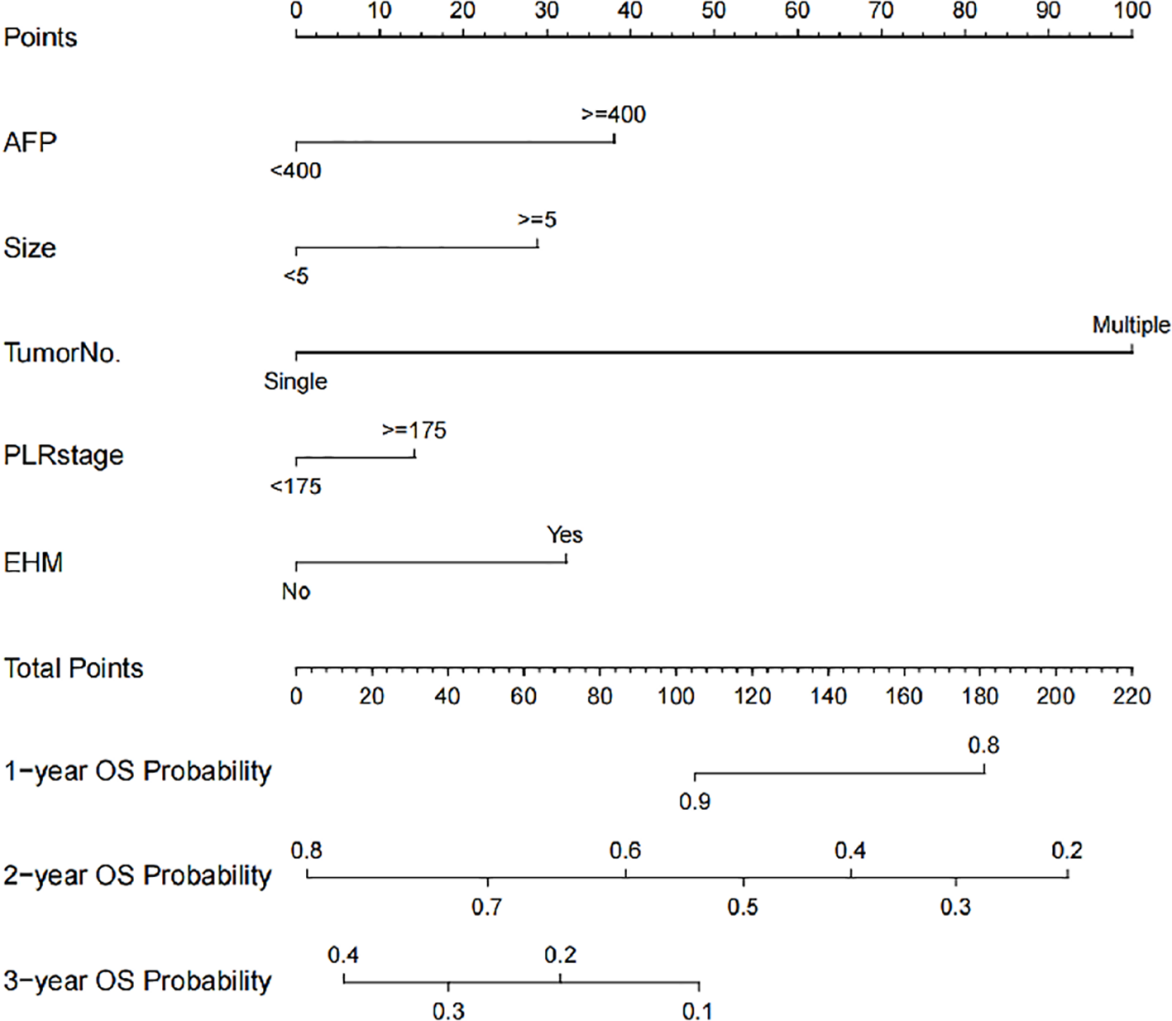
PLANES model to predict OS in training cohort.

**Figure 4:**
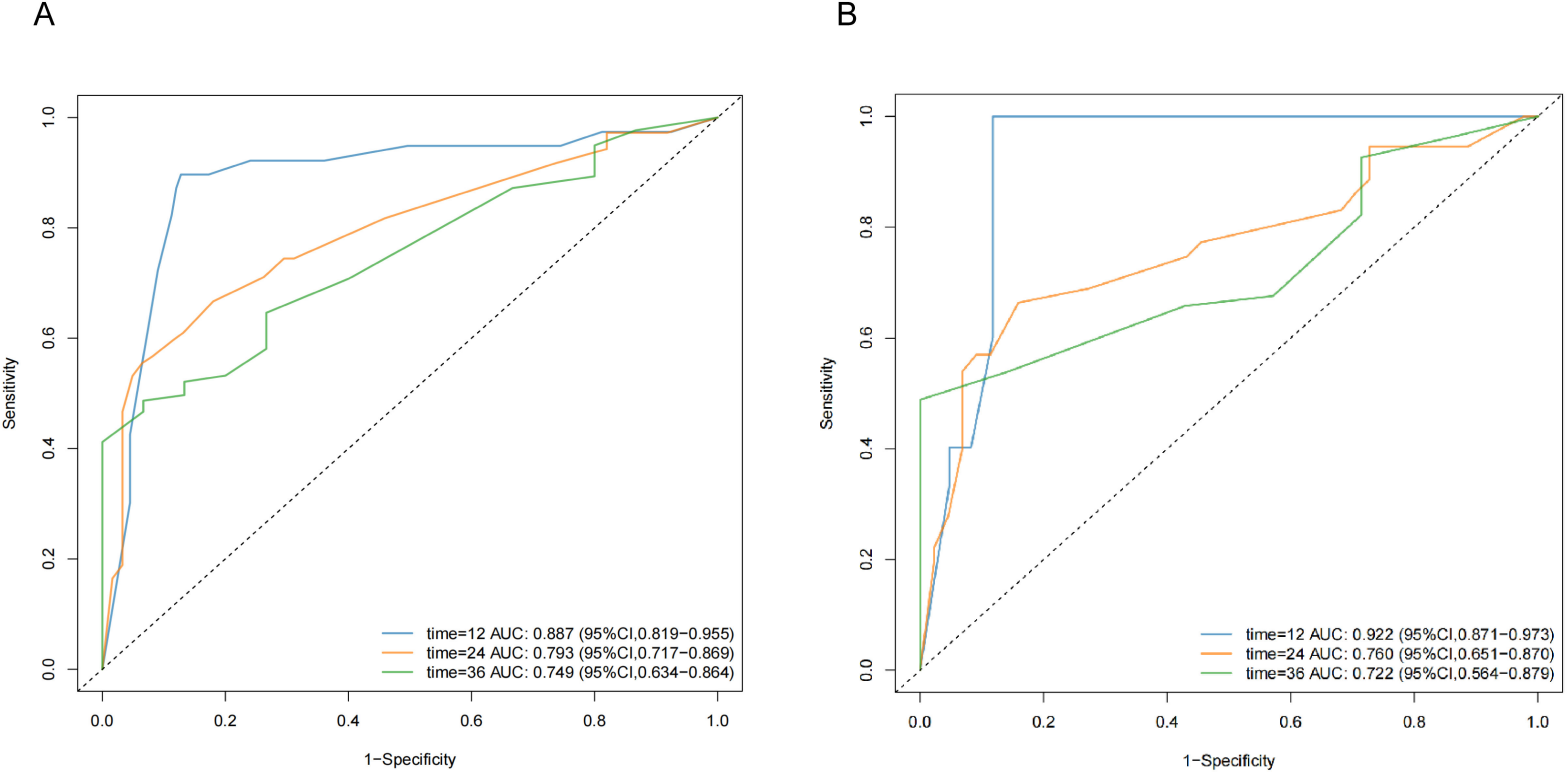
ROC curves for predicting OS in training cohort **(A)** and validation cohort **(B)**.

A nomogram (the PLANES model, which incorporates PLR, AFP level, tumor number, extra-hepatic metastasis, and tumor size) was developed to estimate 1-, 2-, and 3-year OS probabilities (**Figure 3**). The model demonstrated excellent discrimination in the training cohort with AUCs of 0.887 (95% CI 0.819-0.955), 0.793 (95% CI 0.717-0.869), and 0.749 (95% CI 0.634-0.864) for 1-, 2-, and 3-year OS, respectively. External validation yielded AUCs of 0.922 (95% CI 0.817-0.973), 0.760 (95% CI 0.651-0.870), and 0.732 (95% CI 0.564-0.879) at corresponding time points (**Figure 4**). For example, a patient with AFP ≥ 400 ng/ml, maximum tumor size < 5 cm, multiple tumor, PLR < 175 and presence extra-hepatic metastasis would score a total of 170 points (37.5 points for AFP level, 0 points for tumor size, 100 points for tumor number, 0 points for PLR level, and 32.5 points for extra-hepatic metastasis), which the predicted probability of 1-year, 2-year, and 3-year OS was 82.0, 34.0, and 0%, respectively.

**Figure 5:**
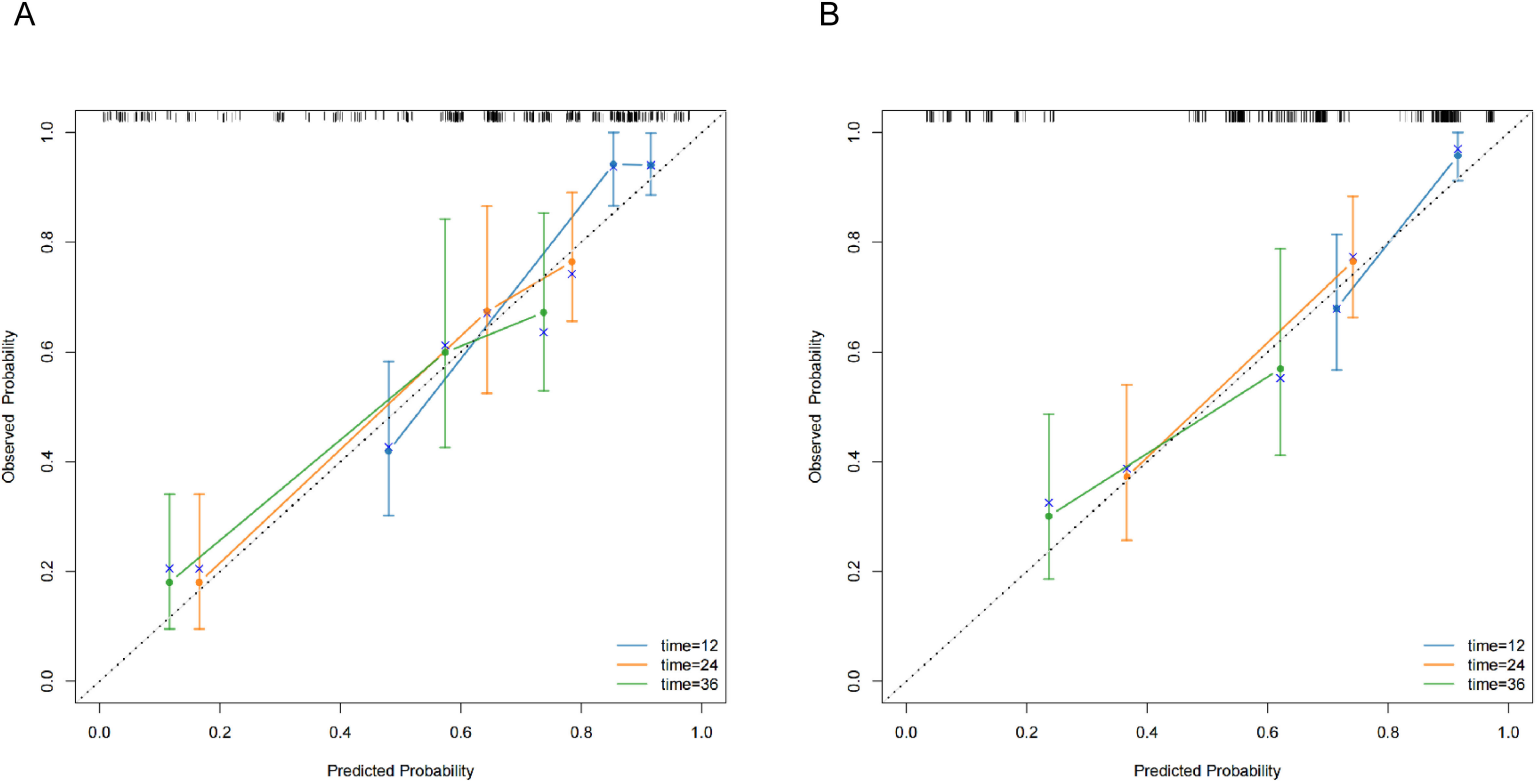
Calibration curves for predicting OS in training cohort **(A)** and validation cohort **(B)**.

**Figure 6:**
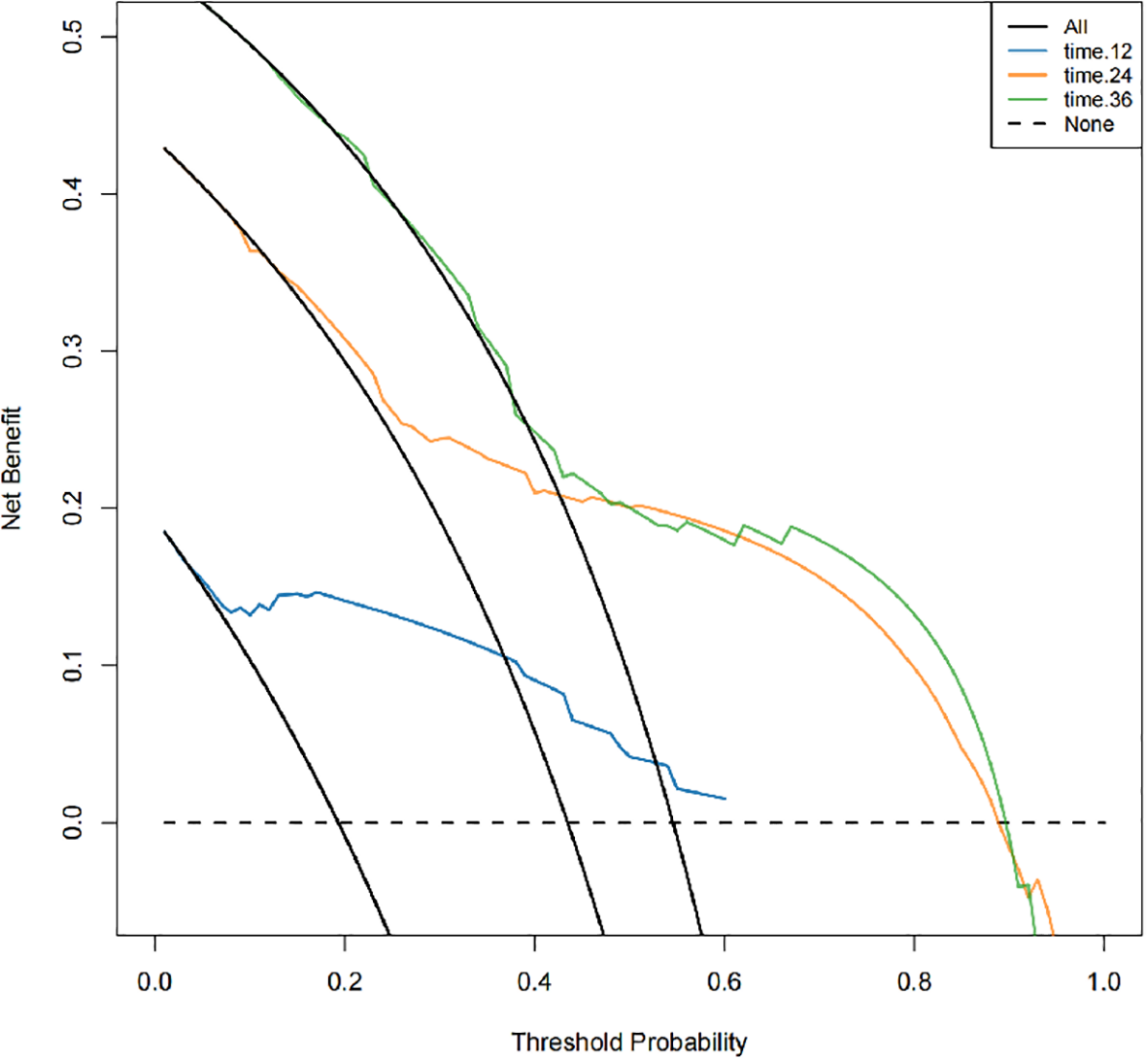
Decision curve analysis for OS in the training cohort. (Dotted line: All patients dead. Black solid line: None patients dead. Color line: Model of nomogram.).

Calibration curves demonstrated excellent concordance between nomogram-predicted and actual observed overall survival probabilities across 1-, 2-, and 3-year timepoints (**Figure 5**). DCA confirmed clinical utility across both cohorts (**Figure 6**).

## 4 Discussion

BCLC staging remains a cornerstone in HCC management. Our study specifically focused on patients with BCLC stage B or C disease, for whom locoregional or systemic therapies are recommended when R0 resection is unattainable. Growing clinical evidence supports triple therapy (TACE + lenvatinib + PD-1 inhibitors) as an emerging standard for uHCC due to its potential to stimulate systemic immune responses (18, 21, 32–34). The principal strength of our study lies in its large real-world cohort with extended follow-up. We report a mOS of 30.8 months—the longest observed to date for this regimen—and an ORR of 60.6% per mRECIST criteria. Nevertheless, therapeutic resistance remains a clinical challenge. Accurate patient stratification for triple therapy benefit is therefore essential, highlighting the demand for reliable prognostic biomarkers.

With advances in basic research on HCC, several prognostic models exist for uHCC treated with locoregional/systemic therapies (35–37). The CRAFITY score (AFP + CRP) predicts outcomes in immunotherapy-treated HCC (38), while Asian cohort analyses identify Child-Pugh grade, BCLC stage, ECOG PS, tumor burden, and local therapy integration as OS determinants (39). Zeng et al.’s TAE score (bilirubin, AFP ≥400 ng/mL, EHM) stratifies triple therapy outcomes (40), and Li et al. highlight early tumor response and conversion surgery as prognostic indicators (41). Our prior work similarly identified pretreatment AFP, portal vein tumor thrombosis (PVTT), tumor number, and maximum diameter as ORR predictors (42). Despite these advances, clinically feasible biomarkers derived from large cohorts remain limited. Our PLANES model addresses this gap by incorporating tumor characteristics and inflammatory markers to identify triple therapy beneficiaries with superior discriminatory power and clinical utility.

Current staging systems (BCLC, CNLC) dichotomize EHM, tumor size, and number to guide management. Tumor burden, quantified by maximum lesion diameter and multifocal involvement, emerged as a significant survival predictor. Elevated tumor burden consistently correlated with adverse clinical outcomes (43–45). Our findings align with prior studies (32, 34, 46), confirming that elevated tumor burden (size >5 cm or multifocality) independently predicts adverse HCC outcomes. EHM similarly portends advanced disease and poor prognosis (17, 47). In present study, we found that patients without EHM showed a significantly longer PFS and OS with triple therapy, which is similar to previous studies (48, 49). A meta-analysis of 54 studies (n=6,187) investigating EHM in ICIs-treated HCC patients revealed reduced ORR without significant PFS or OS impacts (50). This heterogeneity may stem from prognostically distinct EHM subtypes based on metastatic sites. Consequently, our future work will stratify triple therapy outcomes by specific metastasis locations.

Circulating inflammatory factors and serum biomarkers are significant for the progression of HCC. AFP is a specific serum biomarker of HCC and is widely used in HCC management. AFP elevation correlates with poor outcomes through vascular endothelial growth factor upregulation, impaired phagocytosis, and immune evasion (51, 52). Serum AFP values above 200 ng/mL were associated with worse prognosis in the SHARP trail (10). A study established AFP ≥400 ng/mL as an independent risk factor in uHCC patients receiving triple therapy, subsequently developing the TAE prognostic scoring model (40). Hu et al. constructed the CRAFITY prognostic score, integrating AFP and C-reactive protein levels to predict outcomes in HCC patients receiving TACE combined with TKIs and PD-(L)1 inhibitors (53). Moreover, Luo et al. reported that early AFP reduction of >50% could predicts effectiveness of this triple therapy in uHCC patients (54). Present study found that AFP above 400 ng/mL was associated with significantly poor PFS and OS, consistent with previous results (15, 55). The PLR, an inflammation-based prognostic score, effectively predicts outcomes across multiple malignancies including HCC. Multiple studies validate its utility: Wu et al. documented PLR ≥300 correlating with reduced OS/PFS in atezolizumab-bevacizumab-treated uHCC patients (56), while Wang et al. observed inferior survival with elevated PLR in PD-1/bevacizumab cohorts (57). Supporting these findings, a meta-analysis of 24 trials (n=6,318) confirmed pretreatment PLR elevation predicts poorer overall and recurrence-free survival in palliative therapy recipients (50). Nevertheless, data linking PLR specifically to triple-therapy outcomes remain scarce. Consistent with prior evidence, our study confirms that elevated baseline PLR correlates with significantly shorter OS in uHCC patients undergoing triple therapy.

Safety considerations remain paramount. The most common TRAEs are fever, abdominal pain, and liver function impairment related to TACE (58), hypertension, diarrhea, and hand–foot syndrome to TKIs (59), fatigue, and pruritus to ICIs (60), respectively. The combination treatments often increases the TRAEs (61). In the training cohort, 83.8% of patients developed treatment-related adverse events (TRAEs), including grade 3/4 events in 22.7% of cases. Hepatic impairment constituted the most common TRAE, aligning with extant literature. This finding implies that baseline liver function evaluation may guide triple therapy patient selection and necessitates judicious use in individuals with preexisting hepatic dysfunction. Meanwhile, no patient died due to TRAEs in two cohorts. Collectively, this triple therapy demonstrated manageable tolerability with vigilant treatment course monitoring, enabling early adverse event detection and prompt intervention to mitigate severe complications.

There are several limitations in this study except for potential bias due to the nature of the retrospective. First, the follow-up period was relatively short. The median follow-up duration of 25.6 months, while sufficient for capturing a robust number of events for the primary survival analysis, may affect the precision of our longest-term (3-year) survival estimates. Given the short-term follow-up period, subsequent extended observation is required for results verification. Second, this study enrolled patients received various kinds of PD-1 inhibitors. However, this cohort could more representative of the real-world situation, and no differences in the prognostic impact were found in many previous studies (62). Third, some patients experienced progressive, ineffective, or intolerable side effects were changed to second-line or other treatments, contributing somewhat to biased results. Fourth, HBV constituted the primary etiology in this cohort, potentially exhibiting distinct clinicopathological characteristics compared to other causes. Additional, while all participating centers adhered to a standardized TACE protocol to minimize variability, subtle technical differences in embolization endpoints are inevitable in real-world practice. Future studies involving diverse etiologies should assess the nomogram’s generalizability and clinical utility. Additionally, current guidelines lack endorsement of triple therapy as first-line uHCC treatment. Validation through ongoing phase III trials is anticipated.

## 5 Conclusions

TACE combined with lenvatinib and PD-1 inhibitors demonstrates promising efficacy and acceptable toxicity in uHCC. The PLANES nomogram provides a clinically valuable tool for pretreatment prognostication, enabling personalized therapeutic decisions for patients considering triple therapy.

## Data Availability

The raw data supporting the conclusions of this article will be made available by the authors, without undue reservation.
